# Actinobacterial community structure in the Polar Frontal waters of the Southern Ocean of the Antarctica using Geographic Information System (GIS): A novel approach to study Ocean Microbiome

**DOI:** 10.1016/j.dib.2018.02.054

**Published:** 2018-02-23

**Authors:** P. Sivasankar, K. Priyanka, Bhagwan Rekadwad, K. Sivakumar, T. Thangaradjou, S. Poongodi, R. Manimurali, P.V. Bhaskar, N. Anilkumar

**Affiliations:** a Department of Environmental Science, Periyar University, Salem, Tamil Nadu, India; b Centre of Advanced Study in Marine Biology, Faculty of Marine Sciences, Annamalai University, Parangipettai, Tamil Nadu, India; c National Centre for Microbial Resource, National Centre for Cell Science, Pune, India; d Science and Engineering Research Board, Department of Science and Technology (Government of India), India; e National Institute of Oceanography, Dona Paula, Goa, India; f National Centre for Antarctic and Ocean Research, Headland Sada, Vasco-da-Gama, Goa, India

**Keywords:** Antarctica, Marine actinobacteria, Southern ocean, GIS, Polar Frontal waters, Microbiome

## Abstract

*Integration of microbiological data and geographical locations is necessary to understand the spatiotemporalpatterns of themicrobial diversity of an ecosystem. The Geographic Information System (GIS) to map and cataloguethe data ontheactinobacterial diversity of the Southern Ocean waterswas completed through sampling and analysis. Water samples collectedat two sampling stations viz.Polar Front 1(Station 1) andPolar Front 2(Station2)during7^th^Indian Scientific Expedition to the Indian Ocean Sector of the Southern Ocean (SOE-2012-13)were used for analysis. At the outset, two different genera of Actinobacteria were recorded at both sampling stations.Streptomyces was the dominantedwiththehigh score (> 60%), followed by Nocardiopsis (< 30%)at both the sampling stations-Polar Front 1 and Polar Front 2-along withother invasive genera such as Agrococcus, Arthrobacter, Cryobacterium, Curtobacterium,Microbacterium, Marisediminicola, Rhodococcus* and *Kocuria. This data will help to discriminate the diversity and distribution pattern of the Actinobacteria in the Polar Frontal Region of the Southern Ocean waters.Itis a novel approachusefulfor geospatial cataloguing of microbial diversityfromextreme nichesand in various environmental gradations.Furthermore,this research work will act as the milestone for bioprospecting of microbial communities and their products having potential applications in healthcare, agriculture and beneficial to mankind. Hence, this research work would have significance in creating a database on microbial communities of the Antarctic ecosystem.*

**Specifications Table**

**Table:** 

*Subject area*	*Microbiology*

*More specific subject area*	*Antarctic Microbiology and Mapping; Geographic Information Systems (GIS); Ocean microbiome*
*Type of data*	*Graph, figure*
*How data was acquired*	*Through wet laboratory work, geo-database*
*Data format*	*Analyzed*
*Experimental factors*	*Isolation, identification and mapping of actinobacterial data using authentic software/tools*
*Experimental features*	*Mapping of the actinobacterial diversity of the Polar Frontal waters of the Southern Ocean of the Antarctica on GIS platform/ GIS database managed by USGS (USA) and processed using the software ArcGIS 10.1.*
*Data source location*	*Polar Frontal waters of the Southern Ocean of the Antarctica*
*Data accessibility*	*The recorded data is included in this article.*

**Value of the data**

• This is a comprehensive approach to map the actinobacterial diversity of the Southern Ocean of the Antarctica.
• The generated data would be used to build the new database on microbial communities of the Antarctic ecosystem and Earth's Microbiome. It will act as the proxy for new research and planning of Mega Expeditions and subsequent expeditions in future.
• Recorded datasets act as limelight for mapping of the spatially and temporally diversity of marine microbes in near future, to pursue research in the field of microbial biogeography, and to discern microbial diversity.

## 1 Data

### 1.1 Background information and data

Earth's microbiome has its own scientific, social and economic importance. Diverse microbial communities exist in unique habitats possesses versatile characteristics, which can be used for societal benefits and environmental applications. Thus, proper cataloguing of microbial diversity and physiological uniqueness *vis-à-vis* its geospatial location is crucial for the improved harnessing of these microorganisms for their potential applications. In spite of our understanding of the global distribution of microbes, their datasets on spatial variability remain elusive. Hence, there is a need to integrate microbiological data and geographical locations in order to understand the spatiotemporal patterns of the microbial diversity.

With the development of the Geographic Information System (GIS), a meaningful approach can be made to catalogue microorganisms in the context of their geographical position and geological and geochemical habitats. This will help discern the distribution and diversity of the microorganisms in space and time, and visualize these trends at greater spatial scales. In addition, it will also beneficial to the scientific community in near future for research work and support resource management. Mapping of chlorophyll, plankton and marine fishing zones has been done [1], [2], [3], [4] in different locations of the world. Recently, a novel approach of GIS-based microbial mapping was employed to explore the spatial diversity of the microbes especially bacteria from the Neil and Havelock Islands of the Andamans [5]. Identification of bacteria was carried out using 16S rDNA gene sequence analysis. The identified strains were mapped in GIS platform with Landsat 8 image as the base.

However, there are no studies relating to the Remote Sensing (RS) and GIS applications for delineating the spatiotemporal variations of microbes in general and in the Polar Frontal waters of the Indian Ocean sector of Southern Ocean, in particular. Above information states that RS and GIS-based studies are indispensable for mapping the microbial diversity of Antarctica. Polar Frontal waters of the Southern Ocean of the Antarctica are most productive marine environs with diverse biota, including microbes. Hence, the present study was carried out to map the actinobacterial diversity of the Polar Frontal waters of the Southern Ocean of the Antarctica in GIS platform.

### 1.2 Outcome of the research

**Fig. 1:**
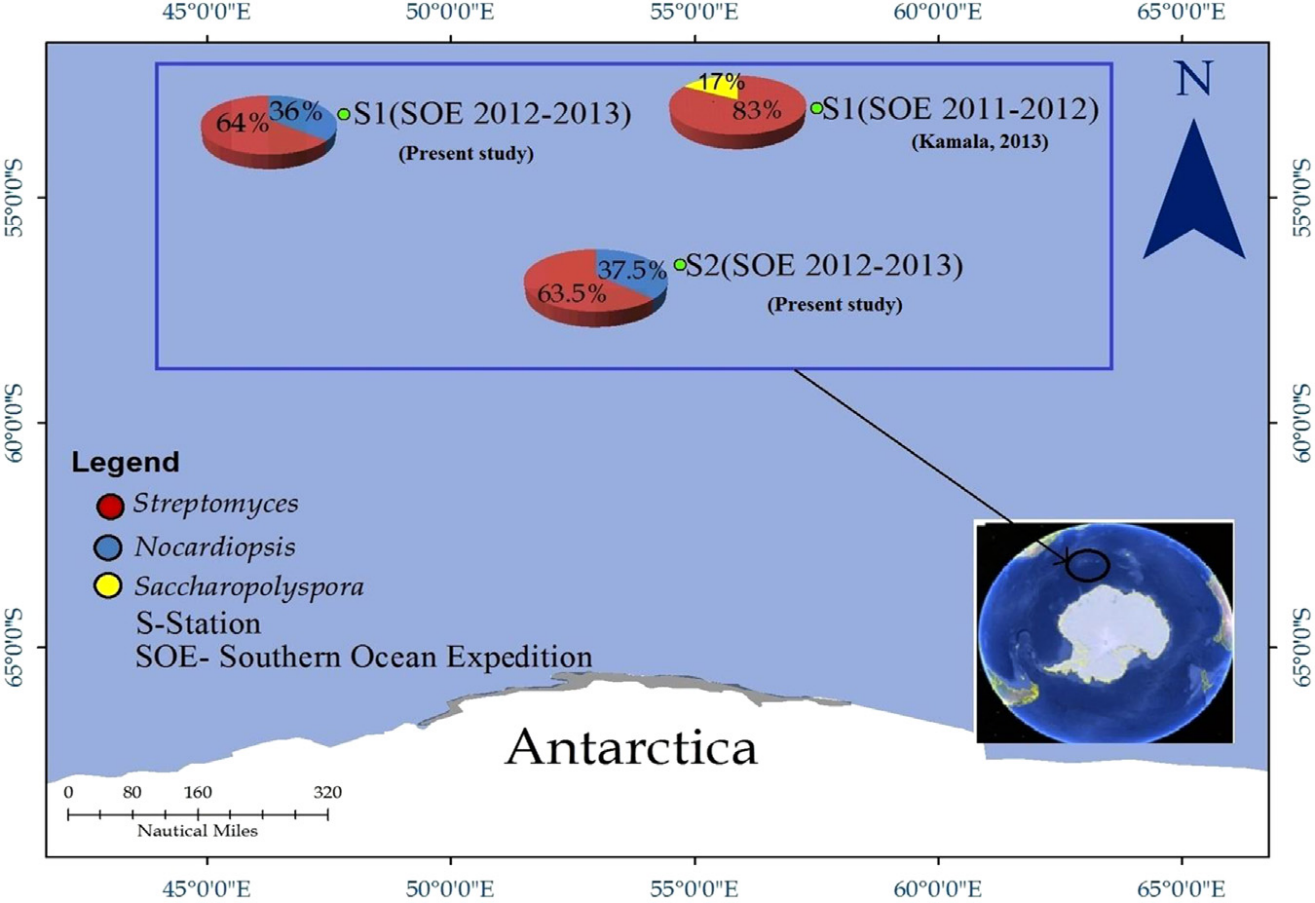
Map showing the cultivable actinobacterial diversity of the Polar Frontal waters of the Southern Ocean of the Antarctica.

**Fig. 2:**
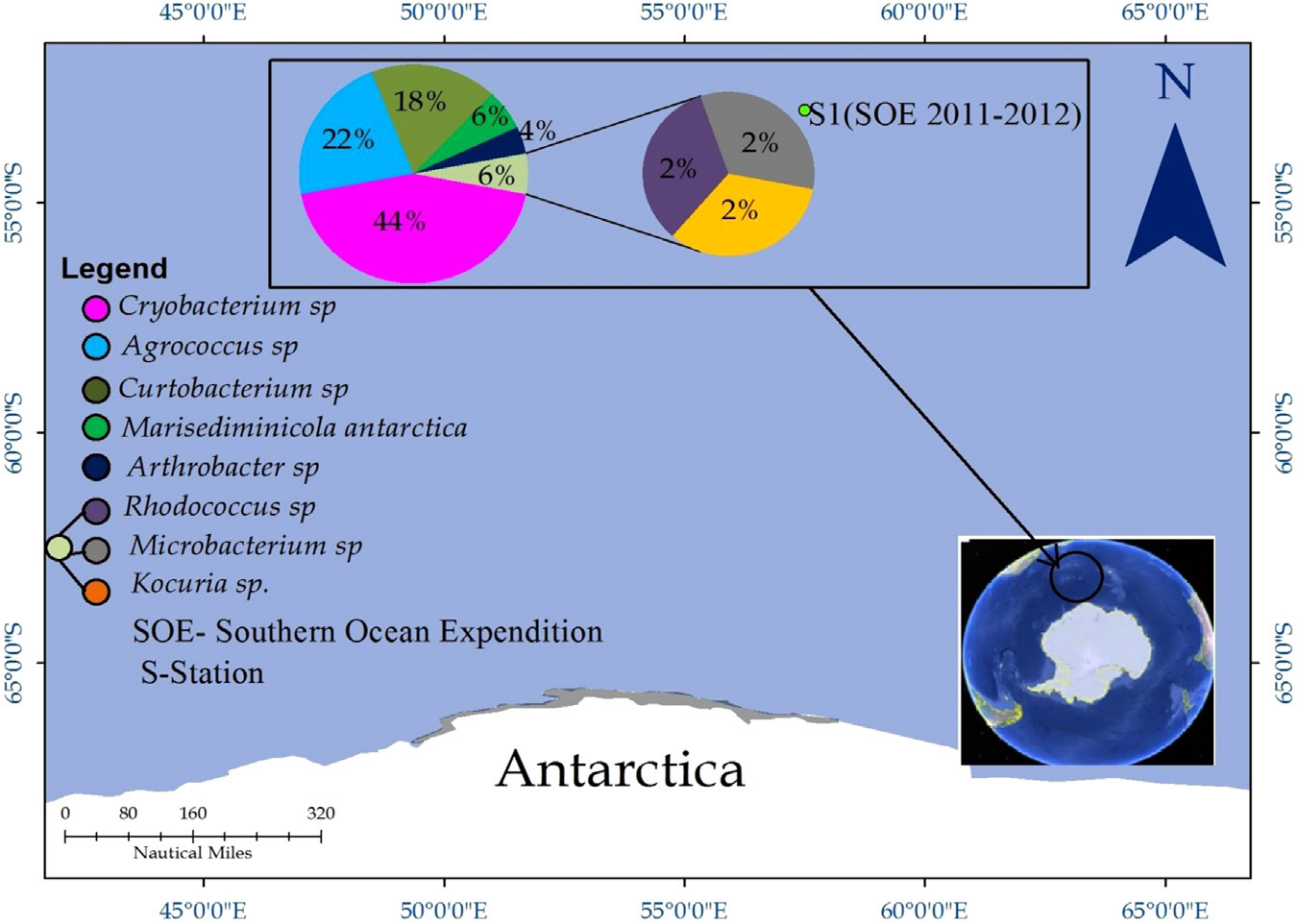
Map showing the metagenomic actinobacterial diversity of the Polar Frontal waters of the Southern Ocean of the Antarctica.

In this actinobacterial microbiome study, an attempt was made to map cultivable actinobacterial diversity in the Polar Frontal waters of the Southern Ocean of Antarctica at two stations viz. sampling station 1 (Polar Front 1) and sampling station 2 (Polar Front 2). Polar Front 1 and Polar Front 2 showed remarkable similarity in existing microbial community where *Streptomyces* was the dominanted by genus (64% at Polar Front 1 and 63.5% at Polar Front 2) followed by *Nocardiopsis* (36% at Polar Front 1 and 37.5% at Polar Front 2) in these sampling days. It is worth mentioning here that we reported the presence of *Streptomyces* (83%) and *Saccharopolyspora* (17%) from the Southern Ocean waters. Thus, temporal and spatial variations are evident in the geographic distribution of Actinobacteria in the Southern Ocean waters (Fig. 1). Based on the metagenomic studies [6], we have further shown that the Southern Ocean waters harboured by other invasive genera such as *Agrococcus, Arthrobacter, Cryobacterium, Curtobacterium, Microbacterium, Marisediminicola, Rhodococcus* and *Kocuria* (Fig. 2).

In Antarctica, RS and GIS have been used to predict the Sea level rise and to prepare the digital elevation model of the South Shetland Islands [7]. A new elevation model of Antarctica was derived from ERS-1 satellite altimetry supplemented with conventional data. This data was used to delineate the ice flow drainage basins across Antarctica, so as to confirm the great uncertainty in the overall contribution of the Antarctic Ice Sheet to recent and future global sea level rise even without a substantial collapse of the West Antarctic Ice Sheet [8].

Gao et al. created an effective means by which the acquired data were analysed for the effective monitoring and mapping of temporal dynamics of the glaciers [9]. The efficacy of glacial erosion since this time was modelled using BEDMAP Antarctic digital elevation data and seismically estimated with offshore sediment volumes to derive a DEM of the pre-glacial topography. Using GIS, sediment was “virtually backstacked” on to the present-day topography under dynamic ice sheet conditions to produce a model of the pre-glacial landscape of the Lambert basin area in East Antarctica; presence of a pre-glacial river valley system under the ice has suggested that the glacial modification of the Lambert region has been modest [10].

From the above account, it can be understood that a comprehensive approach to map the actinobacterial diversity of the Southern Ocean is an imperative need. The present study is an earnest attempt to map the actinobacterial diversity of the Polar Frontal waters of the Southern Ocean of Antarctica, using the GIS approach. It can be added that such mapping of the marine microbes would pave way for the future workers to pursue research in the field of microbial biogeography, to discern microbial diversity, both spatially and temporally. This would greatly help explore the microbial populations from the appropriate marine and coastal locations to find out their potential applications in biotechnology, bioprospecting, biomonitoring and bioremediation. This work is also significant on the score that it helps build a database on microbial communities of the Antarctic ecosystem.

## 2 Experimental design, materials and methods

### 2.1 Study area and sampling

Water samples were collected during the 7^th^ Indian Scientific Expedition to the Indian Ocean Sector of the Southern Ocean (SOE-2012-13) organized by National Centre for Antarctic and Ocean Research (NCAOR), Goa, India. The samples were collected at two stations *viz*., Polar Front-1 (53°07'90"S; 47°48'061"E) and Polar Front-2 (56°29'956"S; 54°41'213"E) using CTD (SEABIRD 911 plus, USA). A minimum of 5 l of the water sample was filtered through 0.22 µm membrane filter. These filter papers were immersed aseptically in pre-sterilized sterile seawater and 20% glycerol stock separately. The sterile seawater loaded with the microbial community was used for immediate isolation of bacteria while 20% glycerol stocks were used isolation of microorganisms over a period of time and stored at -80 ^o^C for long-term use.

### 2.2 Isolation of Actinobacteria from the Polar Frontal waters

The cells retained in the filter paper were dislodged in a 50 ml of sterile seawater and a 100 µl of the sample was spread plated on different selective media (Starch Casein Nitrate Agar (SCNA), International *Streptomyces* Project-2 (ISP2) medium, Antarctic Minimal Medium (AMM), Actinomycete Isolation Agar (AIA) and Arginine-Vitamin Agar (AV), supplemented with nystatin (50 µg/mL) and cycloheximide (50 µg/mL). The inoculated plates were incubated for 60 days at 10 °C for the isolation of psychrophilic Actinobacteria, and at 20 °C for the isolation of psychrotolerant Actinobacteria [11]. After 60 days incubation, the population of the Actinobacteria appeared on agar was counted and expressed in terms of Colony Forming Unit per milliliter(CFU/mL) after calculation.

### 2.3 Identification of the isolated Actinobacterial strains

Isolated actinobacterial strains were identified as per established standard procedures based on their cultural and morphological [12], [13], chemotaxonomical [14] and molecular characteristics [15], [16], [17], [18], [19], [20].

### 2.4 Data collection and processing for mapping the Actinobacteria

GIS data was downloaded from earth explorer (online database managed by USGS) and processed using the ArcGIS (10.1) tool.

### 2.5 Preparation of geo-database

Sequential Earth explorer data was used for the analysis to understand the distribution of Actinobacteria in the Polar Frontal waters of the Southern Ocean of Antarctica. ArcGIS 10.1 tool was used to create final vector maps for spatial mapping. All manually collected GPS points for microbial diversity were plotted as a point location on the spatial map.

## Authors contributions

PS, SP and BR conceived the design and drafted the paper. KP, TT and RM have acquired the satellite data and prepared the map using GIS. KS was participated in the cruise and collected the sample. PVB and NA provided full support to cruise team during the expedition on the cruise for sample collection and transport. BR rectified manuscript, prepared the final draft, reviewed, communicated and revised the paper in this final form.

## Funding

PS and KS are thankful to Department of Science and Technology, Science and Engineering Research Board (DST-SERB) (File No. PDF/2016/003905), and the Department of Biotechnology (DBT), Government of India (File No. BT/PR5426/AAQ/3/599/2012) for financial assistance through the National Postdoctoral Fellowship (N-PDF) Scheme. BR is thankful to University Grants Commission (Government of India) for the financial support in the form of Postdoctoral Fellowship (Grant No. PDFSS-20314-ST-MAH-4350).
